# IL-1R and MyD88 Contribute to the Absence of a Bacterial Microbiome on the Healthy Murine Cornea

**DOI:** 10.3389/fmicb.2018.01117

**Published:** 2018-05-29

**Authors:** Stephanie J. Wan, Aaron B. Sullivan, Peyton Shieh, Matteo M. E. Metruccio, David J. Evans, Carolyn R. Bertozzi, Suzanne M. J. Fleiszig

**Affiliations:** ^1^Vision Science Program, University of California, Berkeley, Berkeley, CA, United States; ^2^School of Optometry, University of California, Berkeley, Berkeley, CA, United States; ^3^College of Chemistry, University of California, Berkeley, Berkeley, CA, United States; ^4^College of Pharmacy, Touro University California, Vallejo, CA, United States; ^5^Department of Molecular and Cell Biology, University of California, Berkeley, Berkeley, CA, United States; ^6^Howard Hughes Medical Institute, University of California, Berkeley, Berkeley, CA, United States; ^7^Graduate Groups in Vision Sciences, Microbiology, and Infectious Diseases & Immunity, University of California, Berkeley, Berkeley, CA, United States

**Keywords:** bacteria, microbiome, murine cornea, AlkDala, DMN-Tre, IL-1R, MyD88, homeostasis

## Abstract

Microbial communities are important for the health of mucosal tissues. Traditional culture and gene sequencing have demonstrated bacterial populations on the conjunctiva. However, it remains unclear if the cornea, a transparent tissue critical for vision, also hosts a microbiome. Corneas of wild-type, IL-1R (-/-) and MyD88 (-/-) C57BL/6 mice were imaged after labeling with alkyne-functionalized D-alanine (alkDala), a probe that only incorporates into the peptidoglycan of metabolically active bacteria. Fluorescence *in situ* hybridization (FISH) was also used to detect viable bacteria. AlkDala labeling was rarely observed on healthy corneas. In contrast, adjacent conjunctivae harbored filamentous alkDala-positive forms, that also labeled with DMN-Tre, a Corynebacterineae-specific probe. FISH confirmed the absence of viable bacteria on healthy corneas, which also cleared deliberately inoculated bacteria within 24 h. Differing from wild-type, both IL-1R (-/-) and MyD88 (-/-) corneas harbored numerous alkDala-labeled bacteria, a result abrogated by topical antibiotics. IL-1R (-/-) corneas were impermeable to fluorescein suggesting that bacterial colonization did not reflect decreased epithelial integrity. Thus, in contrast to the conjunctiva and other mucosal surfaces, healthy murine corneas host very few viable bacteria, and this constitutive state requires the IL-1R and MyD88. While this study cannot exclude the presence of fungi, viruses, or non-viable or dormant bacteria, the data suggest that healthy murine corneas do not host a resident viable bacterial community, or microbiome, the absence of which could have important implications for understanding the homeostasis of this tissue.

## Introduction

Diverse communities of resident microorganisms on host tissues (microbiomes) play important roles in maintaining health and development of a functional immune system (Turnbaugh et al., 2007; Consortium, 2012; Gevers et al., 2012; Thaiss et al., 2016; Blacher et al., 2017). Furthermore, it is becoming increasingly evident that alterations in abundance and diversity of these bacterial constituents are associated with inflammation and disease (Frank et al., 2007; Koeth et al., 2013; Scher et al., 2013; Corrêa et al., 2017; Halfvarson et al., 2017). Much work has been done to decipher the role of microorganisms within the nasal and respiratory tract, oral cavity, urogenital tract, gut and skin (Gillan, 2008; Grice et al., 2009; Ravel et al., 2011; Consortium, 2012; Liu et al., 2015; Mark-Welch et al., 2016). However, the location and role of bacteria that normally colonize the ocular surface, and the implications for ocular health and immunity, is only beginning to be appreciated (Doan et al., 2016; Kugadas et al., 2016).

Standard microbiological culture methods and molecular techniques have both been used to demonstrate a conjunctival microbiome. Culture methods revealed small numbers and infrequent growth of bacteria on the human conjunctiva, typically ∼100 or less colony-forming units (CFU) per swab (Fleiszig and Efron, 1992; Willcox, 2013). However, 16S rRNA gene sequencing suggested far more bacterial genera were present on the conjunctiva than indicated by culture results (Graham et al., 2007; Dong et al., 2011; Doan et al., 2016; Ozkan et al., 2017). Still, significantly fewer bacteria are thought to inhabit the conjunctiva compared to other mucosal surfaces, e.g., ∼0.06 bacteria per cell in the human conjunctiva versus 12 and 16 bacteria per cell in the oral cavity or on the skin, respectively (Doan et al., 2016).

Culture and sequencing methodologies used to demonstrate conjunctival microflora each have limitations. Nucleic acid sequencing does not equate to viable bacteria, and is therefore prone to false-positive results, while culture methods can miss viable microbes, e.g., bacteria with fastidious nutritional requirements or those undergoing stress responses, thereby prone to false-negative results (Oliver, 2005; Epstein, 2013; Kawai et al., 2015). Moreover, the lower biomass at the ocular surface can also lead to false-positive results due to contaminants from the environment or reagents (Schabereiter-Gurtner et al., 2001; Salter et al., 2014). Importantly, neither culture nor sequencing provides spatial information on bacterial location at the ocular surface. To that end, scientific publications often do not appear to distinguish the cornea from conjunctiva in reporting the “ocular surface” microbiome (Dong et al., 2011; Lu and Liu, 2016; Ozkan et al., 2017). Thus, our understanding of the bacterial landscape on the eye remains unresolved.

The cornea of the ocular surface is critical to vision, and unusual among body surfaces in its remarkable resistance to infection. The cornea is protected against colonization by pathogenic bacteria by multiple defenses which were originally thought to be due to blinking, and the antimicrobial and aggregative components of tear fluid (Masinick et al., 1997; Kwong et al., 2007; Evans and Fleiszig, 2013). It is now known that tear fluid plays much more complex roles and that epithelial-expressed mucins, tight junctions and antimicrobial peptides are also involved (McNamara et al., 1999; Yi et al., 2000; Blalock et al., 2007; Kwong et al., 2007; Augustin et al., 2011; Mun et al., 2011; Tam et al., 2012; Evans and Fleiszig, 2013; Li et al., 2017).

Indeed, the opportunistic bacterial pathogen *Pseudomonas aeruginosa* will not colonize the healthy corneal surface. Susceptibility to bacterial adhesion requires the introduction of some form of compromise to the surface epithelium (Alarcon et al., 2011) or to innate defenses such as MyD88-deficiency (Tam et al., 2011). Deliberate inoculation of healthy corneas with *P. aeruginosa* results in rapid clearance of the bacteria without colonization or pathology (Mun et al., 2009; Augustin et al., 2011). Thus, the murine cornea appears inhospitable to *P. aeruginosa*, a bacterium unusual in its capacity to survive in a diverse array of conditions. This could have led to an assumption among some researchers in the field that the cornea is inhospitable to bacteria in general, albeit a notion not actually proven.

The aim of this study was to determine if the healthy mouse cornea hosts a resident bacterial microbiome, and whether it is able to clear other deliberately inoculated bacteria as effectively as *P. aeruginosa*. We utilized a novel approach to overcome obstacles and limitations of traditional culture and sequencing methodologies. An alkDala probe was used to label only viable, metabolically active, bacteria *in situ*. This reagent utilizes the ability of peptidoglycan metabolic enzymes to take up natural and unnatural D-amino acid substrates to insert into the stem peptides of cell wall peptidoglycan if bacteria are metabolically active (Siegrist et al., 2013, 2015; Shieh et al., 2014). Bacteria that have incorporated the probe are then detected using an azide-fluorophore via copper-catalyzed click chemistry, and imaged by fluorescence microscopy (Siegrist et al., 2013). AlkDala labeling has previously only been used to label bacteria *in vitro* (Siegrist et al., 2013, 2015; Shieh et al., 2014). FISH of a universal 16S rRNA gene probe was also used to detect viable bacteria for which peptidoglycan metabolism was absent, inactive, or at a very low level (Vaishnava et al., 2011; Mark-Welch et al., 2016). Results showed that healthy murine corneas are broadly inhospitable to bacteria, a constitutive state requiring the IL-1R and MyD88, and that consequently they lack a resident viable bacterial community, or microbiome.

## Materials and Methods

### Bacterial Strains

*Pseudomonas aeruginosa* (strain PAO1), *Staphylococcus aureus* (isolated from a human corneal infection*)*, and a Coagulase-negative *Staphylococcus* (CNS) species (sp.) isolated from a mouse eyelid (this laboratory) were used in the clearance experiments. CNS sp. were confirmed using standard biochemical diagnostic tests. *P. aeruginosa* strain PAO1 expressing d-Tomato on plasmid p67T1 (PAO1-dtom) was used for experiments to validate bacterial labeling methods (Singer et al., 2010). Inocula were prepared from overnight cultures grown on TSA plates at 37°C for ∼16 h before suspension in phosphate-buffered saline (PBS) to a concentration of ∼10^10^ or ∼10^11^ CFU/mL. PAO1-dtom was grown on TSA supplemented with carbenicillin (400 μg/mL). Viable counts of bacterial suspensions were performed by serial dilution in PBS as needed (typically from 10^-6^ to 10^-9^) and plating in triplicate onto TSA plates, followed by incubation for ∼18 h at 37^o^C to determine CFU.

### Bacterial Isolation and Identification

To culture bacteria from the corneas of wild-type and IL-1R (-/-) mice, the corneal epithelium was collected using the Algerbrush II, placed in 500 μL 0.25% Triton + PBS, then vortexed. An aliquot (100 μL) of lysate was inoculated onto TSA, blood agar, or chocolate agar and incubated at 37°C in aerobic and anaerobic conditions for up to 7 days. Isolated bacterial colonies were identified by direct colony PCR of the 16S ribosomal RNA gene using universal primers P11P (5′-GAGGAAGGTGGGGATGACGT-3′ and P13P (5′-AGGCCCGGGAACGTATTCAC-3′ (Widjojoatmodjo et al., 1994). Reaction mixes (50 μL) were set up as follows: 1X Q5 Reaction Buffer (New England BioLabs), 1X Q5 High GC Enhancer, 200 μM dNTPs, 0.5 μM Forward Primer, 0.5 μM Reverse Primer, and 0.02 U/μL Q5 High-Fidelity DNA Polymerase. A sterile toothpick was used to touch a bacterial colony on an agar plate and inserted directly into the PCR reaction tube. The reaction mixtures were subjected to the following thermal cycling sequence on a Bio-Rad Thermal Cycler: 98°C for 3 min followed by 30 cycles of 98°C for 10 sec, 63°C for 20 sec, 72°C for 45 s, followed by a final extension of 72°C for 2 min. Molecular grade water was included as a negative control, and a known strain of *P. aeruginosa* (PAO1) used as a positive control. Following amplification, samples were examined by electrophoresis in 1% agarose gels in 1× TBE buffer. Amplicons were purified using PureLink^TM^ PCR Purification Kit (Invitrogen) and sequenced at the UC Berkeley DNA Sequencing Facility. Sequences obtained were identified using NIH BLAST Search Tool^1^.

### Mice

All procedures were carried out in accordance with a protocol approved by the Animal Care and Use Committee, University of California, Berkeley. Six to 12 weeks old male and female wild-type C57BL/6 mice (from Charles River or Jackson Laboratory), *mT/mG* knock-in mice (bred in house), IL-1R gene knockout (-/-) mice (bred in house), and MyD88 gene knockout (-/-) mice (bred in house) were used. Corneas of C57BL/6 mice have an area of ∼ 5.3 mm^2^. Both male and female mice contributed to the results since no differences were observed between them. Anesthesia was induced by intraperitoneal injection of ketamine (80–100 mg/Kg) and dexmedetomidine (0.25–0.5 mg/Kg) before inoculation with bacterial suspensions (5 μL). At 1 h, or other times post-inoculation, the anesthetic antidote atipamezole (2.5–5 mg/Kg) was administered, and mice were allowed to recover with food and water. Mice were euthanized by intraperitoneal injection of ketamine (80–100 mg/Kg) and xylazine (5–10 mg/Kg) followed by cervical dislocation. All experiments involved at least three animals per group and were repeated at least twice.

### Ocular Clearance of Bacteria

Wild-type C57BL/6 mice (6–12 weeks) were used. After induction of anesthesia, 5 μL of bacterial inoculum containing ∼10^8^ or ∼10^4^ CFU was applied to the healthy ocular surface. At 24 and 72 h post-inoculation, tear fluid was collected from the ocular surface, and the numbers of viable bacteria determined. Tear fluid was collected by capillary action using a 30 μl glass capillary tube from the lateral canthus after 10 μl of PBS was added. The conjunctiva and cornea were then extracted and homogenized in 500 μl of PBS. Viable bacterial counts were determined by 10-fold serial dilution of the homogenates in PBS from 10^-1^ to 10^-4^ before plating in triplicate onto on TSA plates, followed by incubation for ∼18 h at 37 ^o^C to determine CFU. In other experiments, whole eyes were enucleated and subject to FISH *ex vivo* after inoculation with bacteria as above.

### AlkDala (Alkyne-Functionalized D-Alanine) Labeling

Labeling of live bacteria using an alkyne functionalized D-alanine (alkDala) biorthogonal probe (Siegrist et al., 2013; Shieh et al., 2014) was adapted for use on the murine ocular surface. Enucleated eyes were incubated in a solution of alkDala (10 mM) in Dulbecco’s Modified Eagle Medium (DMEM) at 37°C for 2 h. In other experiments, eyes of IL-1R (-/-) and MyD88 (-/-) mice were first incubated in an antibiotic cocktail of gentamicin (300 μg/mL), ofloxacin (300 μg/mL) and vancomycin (5 mg/mL) in DMEM. After alkDala incubation, all eyes were transferred to pre-chilled 70% EtOH and fixed for 20 min at -20°C. After rinsing, eyes were permeabilized in PBS with Triton-X100 (0.5%) for 10 min with shaking at room temperature (RT), then washed 3 times for 5 min each in PBS with Triton-X100 (0.1%) and BSA (3%) with shaking at RT. Eyes were then transferred to the click-labeling cocktail [in PBS, TBTA (100 μM), CuSO_4_ (1 mM), sodium ascorbate (2 mM), 488 nm azide-fluorophore (10 μM), BSA (0.1 mg/mL)] for 1 h with shaking at RT.

### Fluorescence *in Situ* Hybridization

Enucleated eyes were fixed in paraformaldehyde (2%) for 1 h with shaking at RT. Bacterial hybridization was performed using a universal 16S rRNA gene [Alexa488]-GCTGCCTCCCGTAGGAGT-[Alexa488] (Eurofins Genomics) as previously described (Vaishnava et al., 2011; Mark-Welch et al., 2016). Briefly, fixed eyes were washed in 80% EtOH, 95% EtOH, and then PBS for 10 min each with shaking at RT. Eyes were then placed in a hybridization buffer solution [NaCl (0.9 M), Tris–HCl (20 mM, pH 7.2) and SDS (0.01%)] and incubated at 55°C for 30 min. The probe was added to final concentration of 100 nM and incubated at 55°C overnight. Eyes were then transferred to wash buffer solution [NaCl (0.9 M) and Tris–HCl (20 mM, pH 7.2)] and washed three times for 10 min each with shaking at RT.

### DMN-Tre (4-*N,N*-Dimethylamino-1,8-Naphthalimide-Trehalose) Labeling

A DMN-Tre conjugate was used to label bacteria specific to the Corynebacterineae suborder (Kamariza et al., 2018) in conjunction with alkDala-labeling. Conjunctival tissue, aseptically obtained from healthy mouse eyes, was homogenized in 500 μL of DMEM then centrifuged at 14,000 ×*g* for 2 min. Liquid was aspirated and the pellet suspended in alkDala (10 mM) and DMN-Tre (100 μM) in DMEM and incubated at 37°C for 2 h. Samples were processed as described above except that a 647 nm azide-fluorophore was used in the click-labeling cocktail. Then, 10 μL of conjunctival homogenate was spotted on microscope slide with coverslip on top. Conjunctival tissue pieces with alkDala (647 nm) and DMN-Tre (488 nm) fluorescent labels were imaged using a Nikon ECLIPS Ti microscope with a 60× oil-immersion objective.

### Fluorescein Staining

Eyes were rinsed with PBS after the induction of anesthesia as described above. For wild-type mice, one eye was blotted with a Kimwipe^TM^ tissue paper. Eyes of IL-1R (-/-) mice were not blotted. A drop (5 μL) of fluorescein solution (0.02%) was then added to the ocular surface, and corneal epithelial integrity examined using a slit lamp and confocal microscopy.

### Antimicrobial Activity of Corneal Lysates

The antimicrobial activity of murine cornea epithelial lysates was assessed as previously described (Sullivan et al., 2015). Corneas of wild-type or IL-1R (-/-) mice were left untreated or exposed to 5 μl of *P. aeruginosa* antigens [supernatant of a PAO1 culture (Maltseva et al., 2007)] for 3 h. Corneas were extracted and homogenized in distilled water (two corneas per 350 μL of water) and centrifuged at 14,000 ×*g* for 2 min to remove cell debris and stromal tissue. Crude lysates were confirmed to have equal protein concentration with a BCA (bicinchoninic acid) assay kit (Pierce Biotechnology, Inc., Thermo Fisher Scientific, Rockford, IL, United States). *P. aeruginosa* (∼10^6^ CFU/mL) in lysate or water were incubated in triplicate at 37°C with shaking for 3 h. Viable bacterial counts of samples were then determined by 10-fold serial dilution in PBS as described above, plating in triplicate onto MacConkey Agar, followed by incubation for ∼18 h at 37°C to determine CFU. Percentage survival at 3 h was determined as follows: (viable counts from cell lysates at 3 h/viable counts from distilled water at 3 h) × 100%.

### Confocal Microscopy

Murine eyeballs were imaged *ex vivo* as previously described (Tam et al., 2011). Briefly, eyes were fixed to a 12 mm glass coverslip with cyanoacrylate glue with cornea facing upward. The coverslip with eyeball was placed in a 47 mm Petri dish and filled with PBS to cover the eyeball completely. Confocal imaging was performed using an Olympus FV1000 confocal microscope [Olympus BX615Wi upright microscope with Olympus FluoView 1000 detection system equipped with Laser Diodes (LD) 405, 440, 559, 635, and an Argon Laser 488/515]. The 488 nm laser (emission filter: 500–545 nm) was used for detection of bacteria labeled with alkDala or FISH, or corneas stained with fluorescein, the 559 nm laser (emission filter: 570–670 nm) used for detection of red-fluorescent bacteria (PAO1-dtom) or red fluorescent cell membranes, and the 635 nm laser used to obtain ocular surface reflectance (excitation and emission at same wavelength). Three or more randomly chosen fields of each eye (∼0.04 mm^2^) were imaged from the corneal surface through the entire epithelium in 1.0 μm steps as previously described (Tam et al., 2011). Three-dimensional images were reconstructed from z-stacks using IMARIS software (Bitplane). Images were compared to controls to determine threshold for each emission filter and held constant for all images. Bacteria were identified and quantified using the surface generation feature on IMARIS.

### Statistical Analysis

Data were expressed as mean ± standard deviation (SD). Statistical significance of differences between means was determined using an unpaired Student’s *t*-Test or Mann–Whitney *U*-test for two group comparisons. For three or more groups, the Kruskal–Wallis test was used with Dunnett’s multiple comparison test for *post hoc* analysis. *P*-values < 0.05 were considered significant.

## Results

### AlkDala-Labeling Shows the Absence of Metabolically Active Bacteria on the Murine Cornea

**FIGURE 1:**
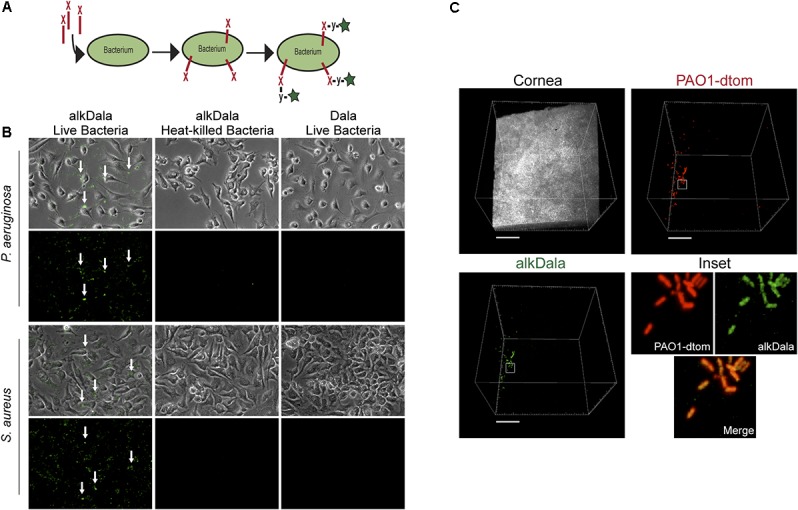
Alkyne-functionalized D-alanine (AlkDala) detects metabolically active bacteria inoculated onto cell culture and the murine cornea. **(A)** Schematic of alkDala (shown as a red X) labeling of bacteria. AlkDala is added to a bacterial suspension to allow incorporation into the cell wall peptidoglycan followed by a copper-catalyzed click-chemistry reaction with an azide-fluorophore (shown as a y-star symbol) to detect bacteria with incorporated probe. **(B)** AlkDala labeling was tested on *Pseudomonas aeruginosa* (PAO1) and *Staphylococcus aureus* (clinical isolate) in the presence of HeLa cells, and labeled all live bacteria (green, arrows) **(Lower Left)**. Heat-killed bacteria were not detected **(Lower Middle)**, nor were live bacteria detected if incubated with D-alanine without an attached alkyne **(Lower Right)**. **(Upper)** show the phase-contrast images of respective fields. **(C)** To determine if bacteria on the murine ocular surface could be labeled with alkDala, whole eyeballs were incubated in ∼10^11^ CFU/mL of red fluorescent *P. aeruginosa* (PAO1-dtom) for 5 h, and then labeled using alkDala (green). The same fields of view are shown with different emission filters. Inset shows zoom of the white box with merge. Scale bar, 50 μm.

To explore whether the cornea hosts a microbiome composed of viable bacteria we used alkDala, a reagent that incorporates into the peptidoglycan of any metabolically active bacterium. Bacteria that have taken up alkDala can then be detected with an azide-fluorophore attached via click chemistry (**Figure 1A**). Prior to use in the mouse eye, these reagents were tested for specificity and efficacy in the presence of host tissue. This was done first using cultured HeLa cells incubated with either *P. aeruginosa* strain PAO1 or *S. aureus* (clinical isolate). The results showed that viable bacteria, but not the inoculated HeLa cells, labeled with the reagents (**Figure 1B**). As expected, heat-killed bacteria or bacteria treated with D-alanine alone (i.e., no alkyne group) did not label (**Figure 1B**). Moreover, environmental fungi did not label with alkDala *in vitro* (Supplementary Figure S1). Next, we explored if the reagent is also able to detect live bacteria in the context of murine corneas. Eyeballs freshly excised from mice were incubated in ∼10^11^ CFU/mL of PAO1-dtom *ex vivo* for 5 h. As shown in **Figure 1C**, bacteria that attached to the corneal surface (visible via dtom), were also detectable by alkDala.

**FIGURE 2:**
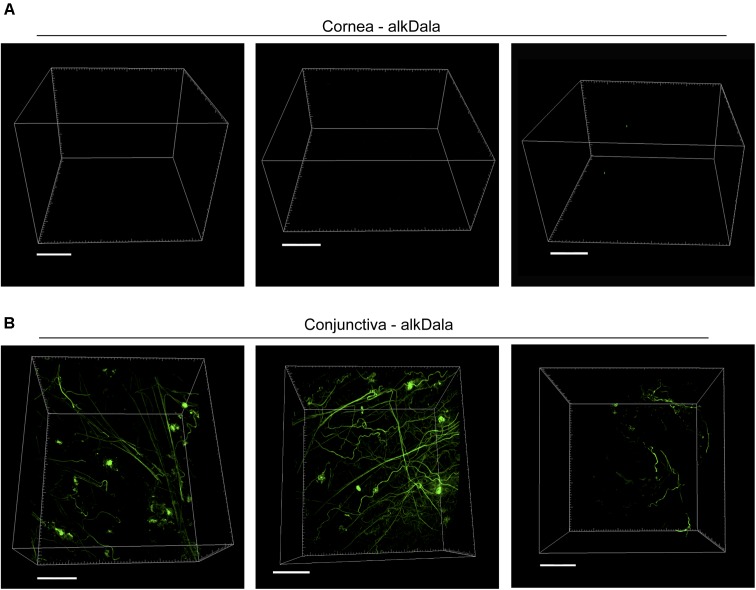
The healthy murine cornea is nearly devoid of viable bacteria. **(A)** AlkDala labeling revealed very few viable bacteria on the healthy corneas of C57BL/6 mice. Representative fields shown from four different labeled corneas (two male and two female mice). **(B)** AlkDala labeling of the conjunctiva of C57BL/6 mice revealed numerous viable bacteria, many in a filamentous form. Representative fields of view are shown from seven different eyes (three male and four female mice). Scale bars, 50 μm.

**FIGURE 3:**
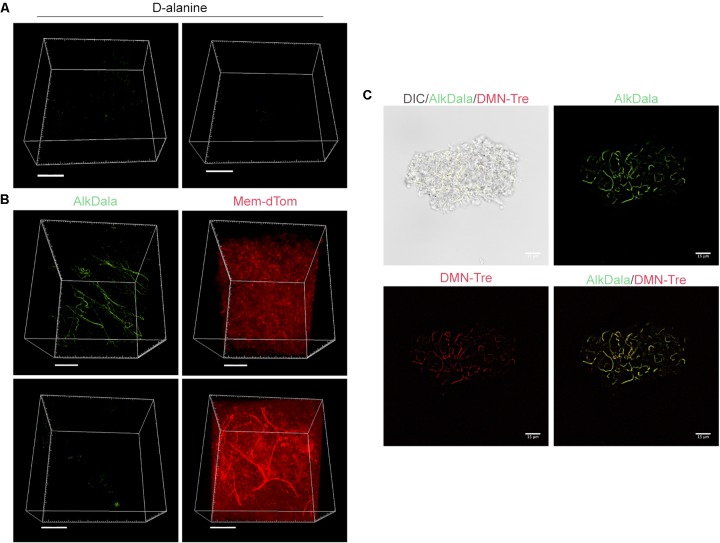
Filamentous structures identified on conjunctiva are not host tissue. **(A)** Mouse eyes incubated in D-alanine, without an alkyne, resulted in no fluorescent labeling. **(B)** Wild-type transgenic mice with fluorescent red cell membranes *mT/mG* knock-in mice (Muzumdar et al., 2007) were used in conjunction with alkDala labeling to determine if the filaments identified on the conjunctiva colocalized with host tissue. Filamentous structures on the conjunctiva **(Upper)** did not colocalize with host cell membranes (denoted Mem-dtom). Conversely, when filament-like structures were present in host tissue, alkDala labeling was not present **(Lower)**. Images shown on the left and right are of the same fields of view with different emission filters. **(C)** Imaging of murine conjunctival epithelial tissue shows that DMN-Tre, a probe specific for Corynebacterineae (red), labeled most of the same conjunctival forms as alkDala (green). All images are from the same field of view with different emission filters.

Having shown that alkDala can distinguish viable bacteria in the context of the eye, we next used it to explore if uninoculated wild-type murine corneas harbored viable bacteria. Three random fields were selected to count the number of visible bacterial forms, the size of each field being ∼0.04 μm^2^. Very few alkDala-labeled bacteria were present on the healthy murine cornea (**Figure 2A**). In contrast, the conjunctiva displayed numerous alkDala-labeled forms (**Figure 2B**), supporting the existence of conjunctival-associated bacteria as previously reported by us, and others, using culture and sequencing methods (Fleiszig and Efron, 1992; Schabereiter-Gurtner et al., 2001; Graham et al., 2007; Dong et al., 2011; Doan et al., 2016). Surprisingly, the alkDala-labeled forms existed in long filamentous and tangled states, not previously reported (**Figure 2B**). Use of D-alanine alone, without an alkyne, confirmed that fluorescent labeling was specific to alkyne incorporation into bacterial cell walls (**Figure 3A**). Furthermore, transgenic mice with red fluorescent cell membranes demonstrated that these alkDala-labeled forms were not host tissue, suggesting that these filamentous structures were microbial (**Figure 3B**).

We hypothesized that the filamentous forms belong to the *Corynebacterium* spp., which are known to inhabit the conjunctiva, and exist in filamentous forms in dental plaque (Mark-Welch et al., 2016; St Leger et al., 2017). To test this, a novel DMN-Tre labeling probe, specific to the Corynebacterineae suborder, was used together with alkDala on murine conjunctival tissue. The DMN-Tre reagent metabolically incorporates into the outer mycomembrane of Corynebacterineae (includes *Mycobacterium* spp. and *Corynebacterium* spp.) as trehalose mycolates (Kamariza et al., 2018). The fluorescence signal is activated upon entry into the hydrophobic mycomembrane. Results showed that the alkDala-labeled filamentous forms (green) tangled in conjunctival tissue also labeled with DMN-Tre (red) (**Figure 3C**) suggesting that they belong to the Corynebacterineae.

### FISH Supports the Absence of Viable Bacteria on Healthy Murine Corneas

To account for the possibility of viable bacteria without peptidoglycan-metabolic processes, or for which peptidoglycan metabolism was inactive or at very low levels, we employed FISH using a universal 16S rRNA gene probe. Unlike alkDala, FISH does not require probe incorporation into bacterial cell wall peptidoglycan, allowing the detection of viable bacteria independently of peptidoglycan metabolism. FISH labeling on the murine cornea was validated by incubating excised whole mouse eyes in ∼10^11^ CFU/mL of red fluorescent *P. aeruginosa* (PAO1-dtom) for 5 h *ex vivo*. After one PBS wash, bacteria remaining on the cornea were detected with FISH (green), and consistently colocalized with red fluorescent *P. aeruginosa* (Supplementary Figure S2).

**FIGURE 4:**
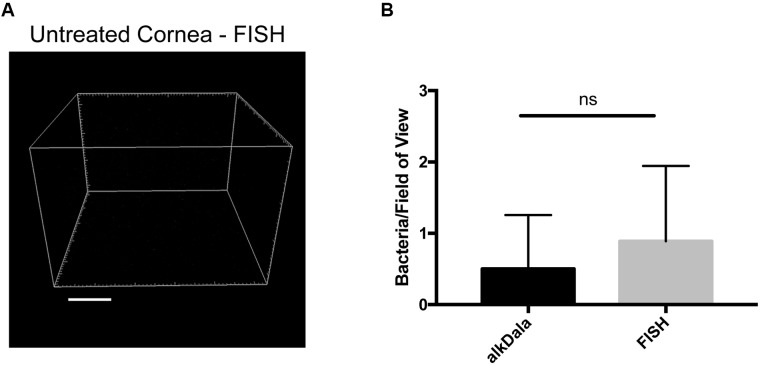
Fluorescence *in situ* hybridization (FISH) labeling confirms that healthy murine corneas are nearly devoid of viable bacteria. **(A)** FISH labeling confirmed C57BL/6 mouse corneas host very few viable bacteria. A representative field of view is shown from four different corneas (two male and two female mice). **(B)** Quantification of viable bacteria on healthy murine corneas using alkDala or FISH expressed as mean ± SD per field of view. One field of view is ∼0.04 mm^2^. ns = there was no significant difference between number of bacteria detected by either method (*P* = 0.40, Student’s *t*-Test).

Fluorescence *in situ* hybridization labeling was then applied to healthy wild-type mouse corneas. Results revealed very few bacterial forms were present on the murine cornea (**Figure 4A**), consistent with previous results with alkDala (**Figure 2A**) and suggesting that the cornea was free of viable bacteria. A comparison of quantitative alkDala-labeling (0.53 ± 0.76 bacteria/field of view, ∼60 bacteria per cornea) with results obtained from FISH labeling (0.88 ± 0.35 bacteria/field of view, ∼100 bacteria per cornea) revealed no significant difference between methods (**Figure 4B**, *P* = 0.40, Student’s *t*-Test). Thus, on rare occasions that bacteria were detected on healthy murine corneas, they mostly existed in viable, metabolically active states.

### Murine Corneas Efficiently Clear Multiple Species of Inoculated Bacteria

**FIGURE 5:**
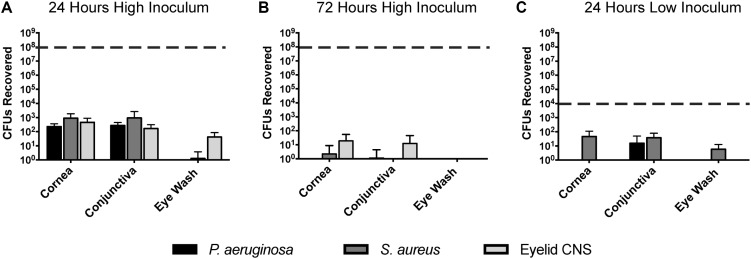
Bacterial clearance from the murine ocular surface. Eyes of C57BL/6 mice were inoculated *in vivo* with *P. aeruginosa, S. aureus*, or a CNS sp. previously isolated from a mouse eyelid, to determine if bacteria could persist on the ocular surface. Data was expressed as the mean ± SD of viable bacteria recovered from the cornea, conjunctiva, or eye wash (see section “Materials and Methods”). The dashed line represents initial inocula. **(A)** After 24 h, the vast majority of inoculated bacteria (∼10^8^ CFU) were cleared from the cornea, conjunctiva, and eye wash. **(B)** By 72 h (3 days), virtually all bacteria were cleared from the ocular surface. **(C)** A lower inoculum (∼10^4^ CFU) resulted in few remaining bacteria after 24 h. In each instance, *P* < 0.05 compared to the initial inoculum (Kruskal–Wallis test, with Dunnett’s multiple comparison).

Previously we reported that *P. aeruginosa* was rapidly cleared from the healthy mouse cornea and tear fluid after deliberate inoculation (Mun et al., 2009). Here, we expanded those studies to determine if *P. aeruginosa*, *S. aureus*, or a murine eyelid commensal (CNS), could gain a foothold in any region of the murine ocular surface after inoculation (**Figure 5**). Thus, healthy wild-type animal eyes were inoculated *in vivo* with ∼10^8^ CFU bacteria, and the number of viable bacteria remaining at multiple locations examined after 24 h. Very few bacteria were recovered from the eyewash/tear fluid of eyes inoculated with the eyelid CNS commensal (∼100 CFU, 6-log reduction compared to inoculum). Bacteria could not be recovered at all from the tear fluid of eyes inoculated with *S. aureus* or *P. aeruginosa*. Corneas and conjunctival tissue each harbored between ∼10^2^ to ∼10^3^ CFU culturable bacteria for all three species, representing a clearance rate of >99.99% of the original inoculum (**Figure 5A**). We next explored if the few bacteria remaining at 24 h could persist on the ocular surface for longer time periods. Thus, experiments were repeated using a 72 h time frame. At this later time point, bacteria were no longer detected in any of the eye washes, and negligible (∼10 CFU) or no bacteria were detected on the cornea and conjunctiva (**Figure 5B**). In case the large inoculum used had activated innate defenses not otherwise involved, experiments were repeated again using a much smaller inoculum (∼10^4^ rather than ∼ 10^8^ CFU). The results revealed even fewer bacteria recovered after 24 h than were recovered after the larger inoculum (**Figure 5C**). No ocular pathology was observed in any of the experiments.

**FIGURE 6:**
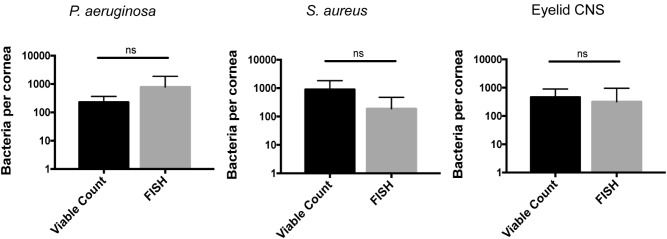
Cornea-associated bacteria remain culturable. Number of bacteria detected per cornea using viable counts compared to FISH labeling at 24 h after uninjured mouse eyes were inoculated *in vivo* with ∼10^8^ CFU of *P. aeruginosa, S. aureus*, or CNS. Data were expressed as the mean ± SD of bacteria identified per cornea. ns = No significant difference was found between methods used (*P* > 0.05, Mann–Whitney *U*-test).

To determine if some additional remaining bacteria had escaped detection by transitioning into a non-culturable state, we also used the FISH label to detect viable bacterial forms. This was done 24 h after inoculation with ∼10^8^ CFU of *P. aeruginosa, S. aureus*, or CNS. In all instances, the number of bacterial forms visible on corneas using FISH was similar to numbers obtained by viable counts (**Figure 6**). These results further illustrate the in-hospitability of the corneal surface to bacterial colonization and show that bacterial clearance from the healthy corneal surface involves complete removal of bacterial forms, not simply neutralization of viable bacteria.

### Corneas of IL-1R (-/-) and MyD88 (-/-) Mice Host Metabolically Active Bacteria

**FIGURE 7:**
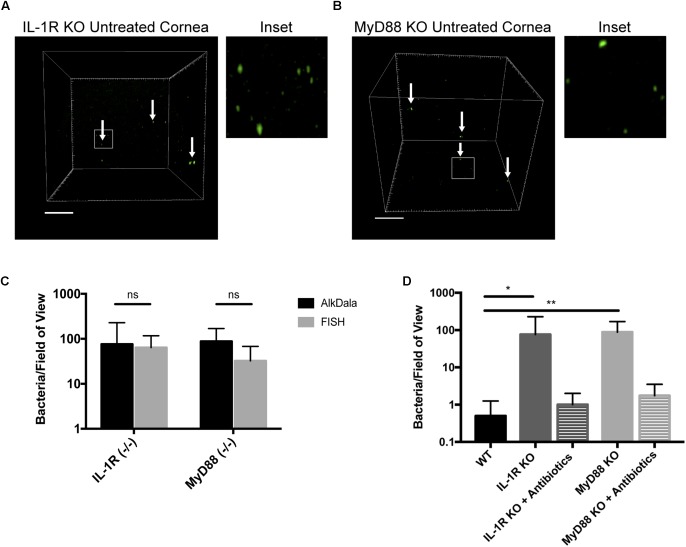
Corneas of IL-1R (–/–) and MyD88 (–/–) C57BL/6 mice harbor significantly more viable bacteria than wild-type. **(A)** AlkDala-labeling revealed many metabolically active bacteria (white arrows) on the corneas of uninjured IL-1R (–/–) mice. A representative field of view is shown from seven different corneas (three male and four female mice). **(B)** MyD88 (–/–) mouse corneas were also colonized with metabolically active bacteria. A representative field of view is shown from six different corneas (two male and four female mice). **(C)** AlkDala and FISH detected similar numbers of viable bacteria on Il-1R (–/–) or MyD88 (–/–) corneas (ns, not significant, Mann–Whitney *U*-test). **(D)** Quantification of viable bacteria detected per field of view using alkDala. Mean ± SD viable bacteria on the murine cornea was significantly higher in IL-1R (–/–) and MyD88 (–/–) mice versus wild-type, ^∗^*P* < 0.05, ^∗∗^*P* < 0.01 (Kruskal–Wallis test with Dunn’s multiple comparison). Antibiotic treatment reduced bacterial detection to wild-type levels for IL-1R (–/–) and MyD88 (–/–) corneas (*P* = 0.88 and *P* = 0.66 versus wild-type, Kruskal–Wallis test with Dunn’s multiple comparison). Scale bar, 50 μm.

Mucosal surfaces are normally colonized by a significant number of commensal microbes, which as an aggregate is termed a microbiome. The above results confirmed the notion that the healthy murine cornea differs in this respect by being virtually clear of viable bacteria. To begin to understand the mechanisms by which the cornea maintains this condition, we tested the hypothesis that innate immune defenses that normally detect and respond to microbes are required. Thus, we explored if the IL-1R was involved using knockout mice, given that it plays an integral role in regulation of innate immunity at mucosal surfaces, including the eye (Pearlman et al., 2008; McDermott, 2009). AlkDala labeling revealed that metabolically active bacteria were present on uninjured corneas of IL-1R (-/-) mice (**Figure 7A**). Since we had previously shown that MyD88 was critical for protecting the murine corneal epithelium against penetration by *P. aeruginosa* (Tam et al., 2011), and MyD88 is an important adaptor molecule for IL-1R signaling, MyD88 (-/-) corneas were also examined. AlkDala labeling revealed that uninjured corneas of MyD88 (-/-) mice also harbored metabolically active bacteria (**Figure 7B**). FISH showed similar numbers of bacteria to alkDala labeling confirming that most detected bacteria on IL-1R (-/-) and MyD88 (-/-) murine corneas were viable (**Figure 7C**). Comparison of bacterial numbers on IL-1R (-/-) mice (76.00 ± 152.19 bacteria/field of view, >10,000 CFU per cornea), MyD88 (-/-) mice (87.87 ± 81.29 bacteria/field of view), with wild-type (<1 bacteria/field of view) revealed significant increases in viable resident bacteria in each gene knockout mouse (*P* < 0.05 and *P* < 0.01 for IL-1R- and MyD88-gene knockout mice respectively, versus wild-type corneas, Kruskal–Wallis test with Dunn’s multiple comparison) (**Figure 7D**). Antibiotic treatment reduced the number of alkDala-detected bacteria to wild-type levels for both IL-1R (-/-) and MyD88 (-/-) mice. For IL-1R (-/-) mice, antibiotics reduced bacterial numbers to 1.00 ± 1.00 bacteria/field of view, and for MyD88 (-/-) mice to 1.75 ± 1.75 bacteria/field of view (*P* = 0.88 and *P* = 0.66, respectively versus WT, Kruskal–Wallis test with Dunn’s multiple comparison) (**Figure 7D**).

Next, we sought to determine which bacterial species inhabited the corneas of these immune-compromised mice. Corneal lysates of wild-type and IL-1R (-/-) mice were inoculated onto various agar media, and cultured bacteria identified by direct colony PCR of 16S rRNA (**Table 1**). Wild-type murine corneas revealed few, if any bacteria, consistent with previous results. Only one genus, *Streptococcus* spp. was identified in one of eight eyes with low bacterial numbers (7 CFU). In contrast, bacteria were isolated from seven of eight IL-1R (-/-) corneas with many more bacterial CFU compared to wild-type (**Table 1**). The most common bacteria identified were CNS spp. (five of eight eyes) and *Propionibacterium* (two of eight eyes). A *Bacillus* spp. was also identified in one eye (**Table 1**). It should be noted that the nature of the bacterial genera colonizing IL-1R (-/-) corneas will likely vary according to environment, e.g., differences between animal care facilities would likely affect culture results. Moreover, the limited number and diversity of bacteria isolated in the present study demonstrates the limitations of viable culture techniques and emphasizes the need for multiple methods when characterizing bacterial communities.

**Table 1:** Bacteria identified on the corneas of wild-type and IL-1R (-/-) mice.

	Wild-type		IL-1R (-/-)	
	CFUs/ cornea	Bacteria identified	CFUs/ cornea	Bacteria identified
Eye 1	7	*Streptococcus*spp.	130	*Staphylococcus* spp. (CNS)
Eye 2	0	–	354	*Staphylococcus* spp. (CNS)
Eye 3	0	–	179	*Staphylococcus* spp. (CNS)
Eye 4	0	–	257	*Staphylococcus* spp. (CNS)
Eye 5	0	–	48	*Bacillus* spp., *Staphylococcus* spp. (CNS)
Eye 6	0	–	3	*Propionibacterium* spp.
Eye 7	0	–	13	*Propionibacterium* spp.
Eye 8	0	–	0	–

### IL-1R (-/-) Corneas Exhibit Epithelial Junction Integrity but Reduced Antimicrobial Activity

**FIGURE 8:**
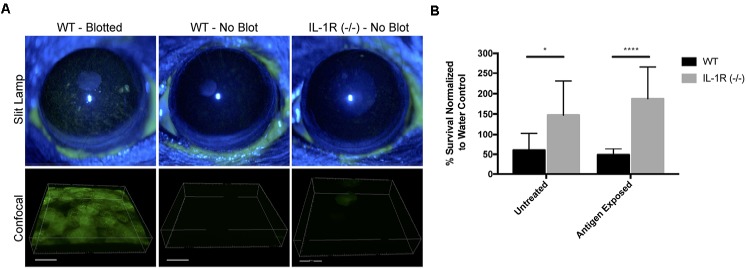
Uninjured IL-1R (–/–) murine corneas do not exhibit fluorescein staining, but show reduced antimicrobial activity. **(A)** After tissue paper blotting, wild-type (WT) corneas were susceptible to fluorescein staining suggesting the disruption of epithelial integrity. Non-blotted (uninjured) WT and IL-1R (–/–) corneas showed little or no fluorescein staining. **(B)** IL-1R (–/–) corneal epithelial lysates showed reduced antimicrobial activity against *P. aeruginosa* strain PAO1 compared to WT lysates. A similar result was obtained in a separate set of experiments in which corneas were exposed to bacterial antigens prior to lysate production. ^∗^*P* < 0.05, ^∗∗∗∗^*P* < 0.0001 (Students *t*-Test).

A potential mechanism for microbial colonization of corneas in knockout mice would be if there was disruption of epithelial tight junctions, which would reduce epithelial barrier function and loss of cell polarity, thereby enabling bacterial colonization of surface epithelial cells (Fleiszig et al., 1997; Alarcon et al., 2011; Tam et al., 2011). To explore that possibility, uninjured corneas of IL-1R (-/-) mice were treated with fluorescein, and compared to corneas of wild-type mice, either uninjured or blotted with a Kimwipe^TM^ to induce superficial injury (Tam et al., 2011). While extensive fluorescein staining was observed in wild-type corneas after superficial injury, uninjured corneas of IL-1R (-/-) mice were similar to uninjured wild-type with little or no staining (**Figure 8A**), suggesting epithelial tight junctions were intact.

Previously, we showed that MyD88 (-/-) mouse corneas also resist corneal staining (Tam et al., 2011), but that corneal lysates from these corneas have reduced antimicrobial activity compared to wild-type (Sullivan et al., 2015). Thus, we examined lysates of IL-1R (-/-) corneas and found that they too were significantly less antimicrobial (2.46-fold) against *P. aeruginosa* PAO1 compared to wild-type mice (*P* < 0.05, Student’s *t*-Test) (**Figure 8B**). Prior exposure of the mouse corneas to bacterial antigens did not impact that outcome, with IL-1R (-/-) lysates remaining less antimicrobial (3.85-fold) than wild-type (*P* < 0.0001, Student’s *t*-Test) (**Figure 8B**).

## Discussion

The ocular surface is constantly exposed to a diverse array of microbes from the environment. In this study, we addressed the question of whether commensal bacteria can inhabit the cornea, similar to the conjunctiva and other mucosal tissues. To do this, we applied metabolic labeling of peptidoglycan using an alkDala probe to directly detect viable bacteria *in situ* using confocal imaging (Vaishnava et al., 2011; Siegrist et al., 2013). Used in conjunction with FISH to identify viable bacteria independently of peptidoglycan metabolism, alkDala has an advantage over traditional culture or 16S rRNA gene sequencing, which are indirect methods with potential for numerous false-negative and false-positive results, respectively. Our results show that the healthy murine cornea is generally inhospitable to bacteria, a condition that requires constitutive function of the IL-1R and MyD88. The results also suggest that, in contrast to the murine conjunctiva and other mucosal surfaces, the healthy murine cornea does not support a resident viable bacterial community (or microbiome).

Having ruled out a natural microbiome in wild-type mice, we explored if we could establish one through deliberate inoculation. We tested pathogens of both Gram-types, and a commensal from the mouse eyelid. These were each rapidly cleared from all regions of the ocular surface (cornea, conjunctiva, and tears), the timing dependent on the initial inoculum size, and the methods used ruled out the possibility that the bacteria had transitioned into a non-culturable viable state. These results also support the notion that the cornea is an inhospitable environment for bacteria.

The results showed that the gain of bacterial colonization in IL-1R and MyD88 (-/-) corneas did not correlate with a loss of barrier function to fluorescein, but was instead associated with a lack of antimicrobial activity in corneal lysates (Sullivan et al., 2015). Thus, local antimicrobial activity is a potential mechanism by which the wild-type cornea maintains its amicrobiomic status. Indeed, IL-1β and toll-like receptor agonists are known to regulate the expression of various antimicrobial peptides, including human beta-defensin 2 (hBD2) and the cathelicidin LL-37, by human corneal epithelial cells (McDermott et al., 2003; Redfern et al., 2011). Possibly related, the murine equivalent of hBD2 (mBD3) is involved in the ocular surface clearance of *P. aeruginosa* after deliberate inoculation under healthy conditions (Augustin et al., 2011). Other potential mechanisms include ocular surface mucins, that can inhibit bacterial adhesion to the cornea (Fleiszig et al., 1994). While we recently showed that glycosylation patterns on the murine ocular surface can be dependent on IL-1R (Jolly et al., 2017), that was not the case for MyD88, and did not necessarily influence bacterial adhesion. Thus, any role for surface glycosylation in maintaining the amicrobiomic status of the cornea is likely to be complex. Our laboratory has also recently shown that uninjured IL-1R (-/-) mouse corneas were significantly more susceptible to *P. aeruginosa* adherence after challenge, and that protection of the cornea against *P. aeruginosa* adhesion involved IL-1R associated with both corneal epithelial cells and CD11C+ cells (Metruccio et al., 2017). Similar mechanisms may play a role in constitutively keeping the cornea free of a bacterial microbiome. It has also been shown that MyD88 (-/-) murine corneas exhibited reduced constitutive levels of cytokines, chemokines, and the matrix metalloproteinase MMP-9 (Reins et al., 2017), which may also contribute to allowing bacteria to colonize the corneas of these mice. Thus, further studies will be needed to delineate factors downstream of IL-1R and MyD88 critical for constitutively keeping the healthy cornea free of resident bacteria under normal conditions.

Contrasting with the cornea, the conjunctiva appeared to be colonized by metabolically active bacteria. Conjunctival-associated alkDala-labeled bacteria appeared mostly as filamentous forms. Controls determined that this labeling was specific to alkDala, and that the filamentous structures did not co-localize with host structures, suggesting that they were indeed microbial. Subsequent imaging experiments showed that DMN-Tre, a probe specific for Corynebacterineae (e.g., a bacterial suborder including *Mycobacterium* and *Corynebacterium* spp.) labeled the same conjunctival filamentous forms as alkDala providing more evidence of their identity. This result was perhaps not surprising since the human conjunctiva is well known to support CNS spp., *Corynebacterium* spp., and *Propionibacterium* spp. (Turnbaugh et al., 2007; Dong et al., 2011; Doan et al., 2016). Moreover, in mice, resident conjunctival-associated bacterial flora contribute to protective ocular immune responses via IL-1β-dependent mechanisms, and local antibiotic treatment reduces corneal immune responses to *P. aeruginosa* infection after scarification injury (Kugadas et al., 2016). Another recent study, using a similar infection model, also implicated resident conjunctival *Corynebacterium mastitidis* in protecting murine corneas from *Candida albicans* and *P. aeruginosa* via IL-17-driven mucosal immune responses (St Leger et al., 2017). In our study, we detected the filamentous bacterial forms on the conjunctiva of mice obtained from both Charles River and Jackson Laboratory. However, we were unable to culture these filamentous bacteria from the conjunctiva of mice used in our study, nor identify *C. mastitidis* from the conjunctiva of mice from Jackson Laboratory, or from other mice in our facility. Further studies will be needed to identify the filamentous bacterial forms and determine their role in ocular surface homeostasis or immune responses.

Many different types of bacteria, including *Corynebacterium* spp., become filamentous when encountering stressful environments, such as in the presence of antibiotics or low nutrients (Wright et al., 1988; Justice et al., 2006, 2008). Possibly, adoption of this morphology represents a deliberate strategy to avoid removal from the ocular surface. For example, filamentation can be used by bacteria to avoid phagocytosis (Horvath et al., 2011). The conjunctiva is not the only place where filamentous bacteria have been identified. Indeed, filamentous *Corynebacterium* spp. are a part of dental plaque and segmented filamentous bacteria (SFB) are commensal inhabitants of the gut (Farkas et al., 2015; Schnupf et al., 2015; Mark-Welch et al., 2016). Unlike other commensals in the gut, SFB are the only bacteria that directly interact with epithelial cells and have been shown to play an important role in modulating the host immune system (Talham et al., 1999; Vaishnava et al., 2011; Farkas et al., 2015).

Some bacteria respond to adverse environmental conditions by entering into a physiological state in which they remain viable, but are not culturable using standard laboratory methods (Oliver, 2010). These are referred to as VBNCs. Since VBNCs retain metabolic activity (Ramamurthy et al., 2014), they would be expected to label with the universal 16S rRNA gene probe used for FISH in our study. While further VBNC-specific detection methodologies would be needed to conclusively exclude VBNCs, our data suggest that they are not present on the healthy murine cornea.

A *caveat* to the present study, is that we cannot exclude the presence of fungi, viruses, or non-viable bacteria on the corneal surface since alkDala did not label these microbes in our studies. Moreover, FISH may not have detected non-viable or dormant bacteria, e.g., if ribosome content was low. Nevertheless, the results of this study support the conclusion that the healthy murine cornea contrasts with the conjunctiva in lacking a resident viable bacterial microbiome. Demonstrating that healthy murine corneas do not host a resident viable bacterial community, commonly present on the conjunctiva, or that are part of other microbiomes, addresses a long-standing knowledge gap in the field, and provides a foundation for a better understanding of corneal homeostasis and disease pathogenesis at the ocular surface. The details of how MyD88 and IL-1R constitutively modulate the absence of a bacterial microbiome on the cornea, and how this is impacted by the environment at the ocular surface, remain to be determined.

## Author Contributions

SW, AS, and PS conducted the experiments. SW, AS, PS, MM, DE, CB, and SF contributed to the data analysis, writing the manuscript, and research design.

## Conflict of Interest Statement

The authors declare that the research was conducted in the absence of any commercial or financial relationships that could be construed as a potential conflict of interest.

## Footnotes

1 https://blast.ncbi.nlm.nih.gov

## Abbreviations

AlkDala: alkyne-functionalized D-alanine
CFU: colony forming units
DMN-Tre: 4-*N,N*-dimethylamino-1,8-naphthalimide-trehalose
FISH: fluorescence *in situ* hybridization
IL-1R: interleukin-1 receptor
MyD88: myeloid differentiation primary response gene 88
TSA: trypticase soy agar
VBNC: viable but not culturable
