# Supplementary figures and images for: IL-1R and MyD88 Contribute to the Absence of a Bacterial Microbiome on the Healthy Murine Cornea
Source: Front Microbiol. 2018 May 29;9:1117. doi: 10.3389/fmicb.2018.01117 (PMC5986933; doi:10.3389/fmicb.2018.01117)

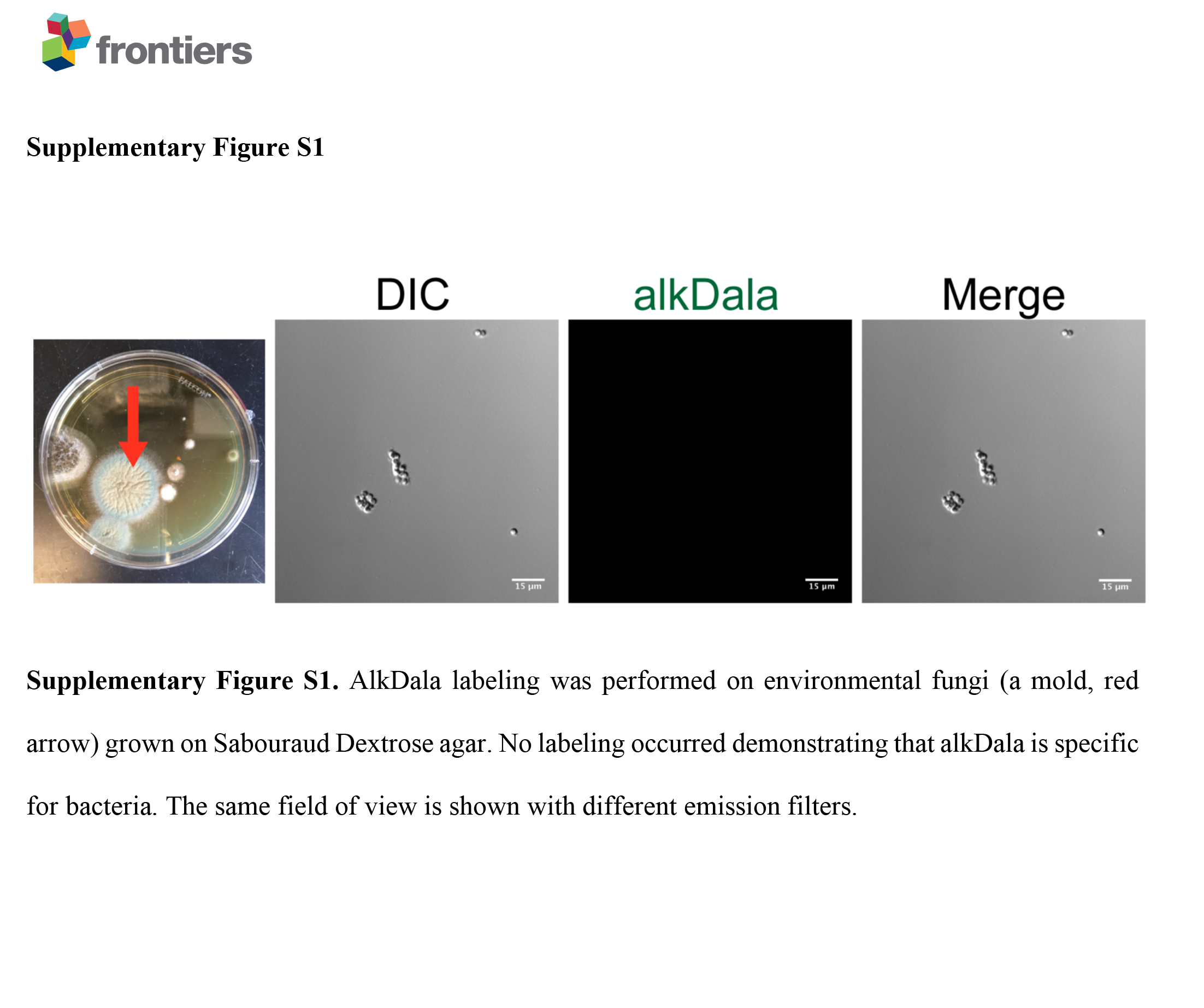

Supplement: Supplementary file 1 [file Image_1.tif]

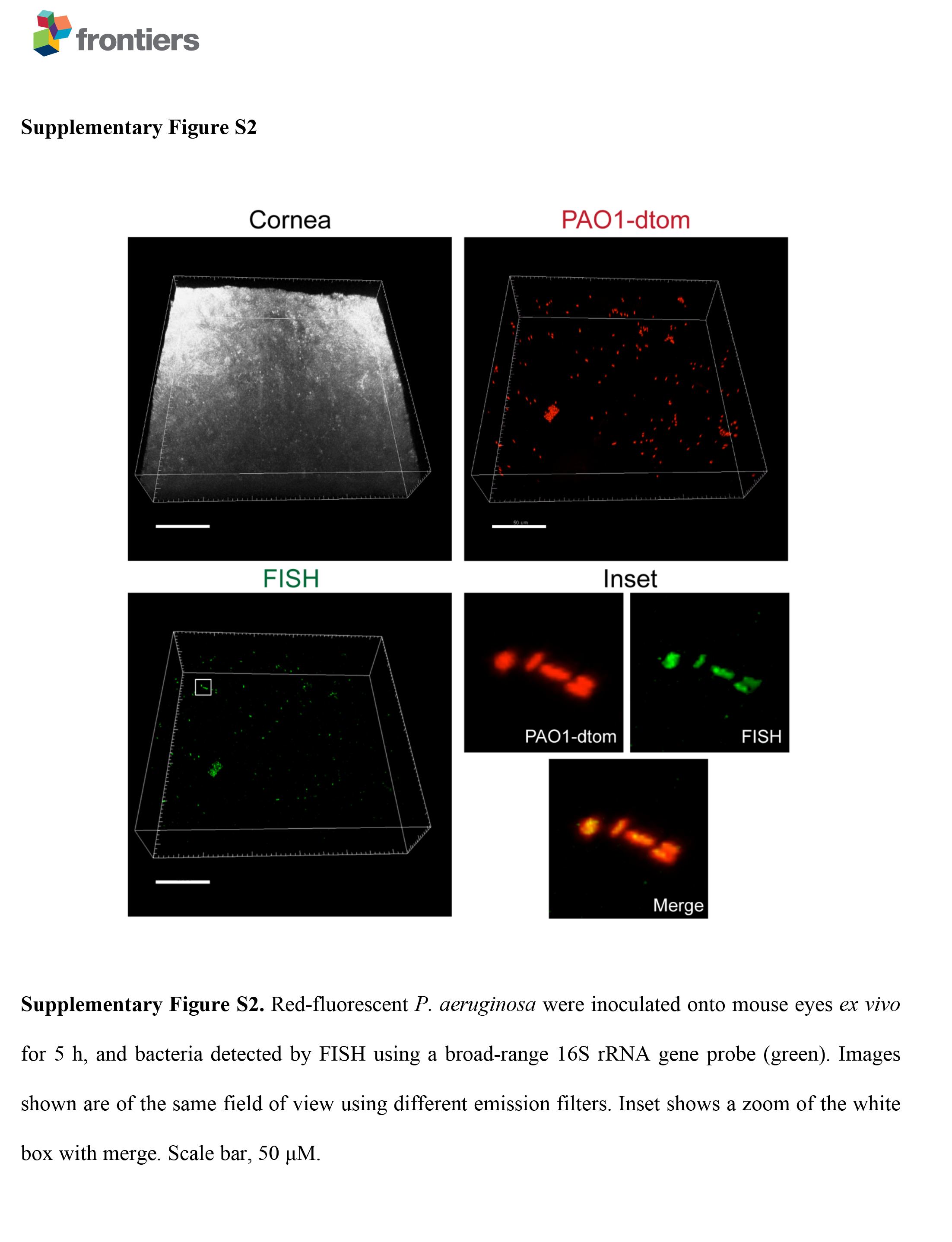

Supplement: Supplementary file 2 [file Image_2.tif]
